# Efficient attribute-based strong designated verifier signature scheme based on elliptic curve cryptography

**DOI:** 10.1371/journal.pone.0300153

**Published:** 2024-05-09

**Authors:** Rui Ma, Linyue Du

**Affiliations:** 1 Xingzhi College, Zhejiang Normal University, Jinhua, China; 2 Personnel Department, Zhejiang Normal University, Jinhua, China; Victoria University, AUSTRALIA

## Abstract

In an attribute-based strong designated verifier signature, a signer who satisfies the access structure signs the message and assigns it to a verifier who satisfies the access structure to verify it, which enables fine-grained access control for signers and verifiers. Such signatures are used in scenarios where the identity of the signer needs to be protected, or where the public verifiability of the signature is avoided and only the designated recipient can verify the validity of the signature. To address the problem that the overall overhead of the traditional attribute-based strong designated verifier signature scheme is relatively large, an efficient attribute-based strong designated verifier signature scheme based on elliptic curve cryptography is proposed, as well as a security analysis of the new scheme given in the standard model under the difficulty of the elliptic curve discrete logarithm problem (ECDLP). On the one hand, the proposed scheme is based on elliptic curve cryptography and uses scalar multiplication on elliptic curves, which is computationally lighter, instead of bilinear pairing, which has a higher computational overhead in traditional attribute-based signature schemes. This reduces the computational overhead of signing and verification in the system, improves the efficiency of the system, and makes the scheme more suitable for resource-constrained cloud end-user scenarios. On the other hand, the proposed scheme uses LSSS (Linear Secret Sharing Schemes) access structure with stronger access policy expression, which is more efficient than the "And" gate or access tree access structure, making the computational efficiency of the proposed scheme meet the needs of resource-constrained cloud end-users.

## 1. Introduction

In the modern information society, digital signature technology has been widely used in various fields, and it is an important tool to ensure data reliability and achieve authentication. Digital signature technology has practical applications in the commercial, financial, and military sectors, especially in e-trade, e-checks, e-shopping, e-publishing, and intellectual property protection.

In traditional standard digital signatures, anyone is able to verify the validity of a signature. However, in many applications where the identity of the signer needs to be protected or the public verifiability of the signature avoided, only the designated recipient of the signature can verify the validity of the signature. For example, in electronic voting, the voter wants to protect the privacy of his or her identity, and only his or her designated verifier can confirm that the signature is valid. But the verifier cannot prove that validity to any third party, and even if the verifier publishes his or her private key, it does not allow the third party to trust that the signature was indeed signed by the voter. To solve this problem, Jakobsson et al. [1] first introduced the concept of Designated Verifier Signature (DVS) in Eurocrypt 1996. However, if a third party intercepts the signature message during message transmission, it can be determined that the recipient did not receive the signed message. It can still be determined that the signature was generated by the signer. Therefore, Saeednia et al. formally proposed the strong designated verifier signature (SDVS) scheme in the literature [2], which requires the participation of the verifier’s private key in the verification algorithm to complete the verification, thus ensuring the private nature of the verification. Strong designated verifier signature (SDVS) provides higher security than designated verifier signature (DVS), and at the same time, better protects the privacy of the signer. This traditional strong designated verifier signature (SDVS) is all about a signer generating a signature that is assigned to a verifier for verification. However, in practical application scenarios, multiple signatures that satisfy certain conditions or certain attributes are used, assigned to multiple verifiers who satisfy certain conditions or certain attributes to verify the signature. For example, in electronic bidding, bids are sensitive information and are not expected to be freely disseminated. It is well suited to use a strong designated verifier signature (SDVS) for signing. By designating agents with certain qualifications as validators, these agents can confirm the validity of the bid to the bid evaluation experts and other necessary persons, and the bidder himself can confirm to the agents and other necessary persons that the bid is his. Other than this, no one else can judge the validity of the tender, much less confirm the validity of the tender to third parties.

**Fig 1:**
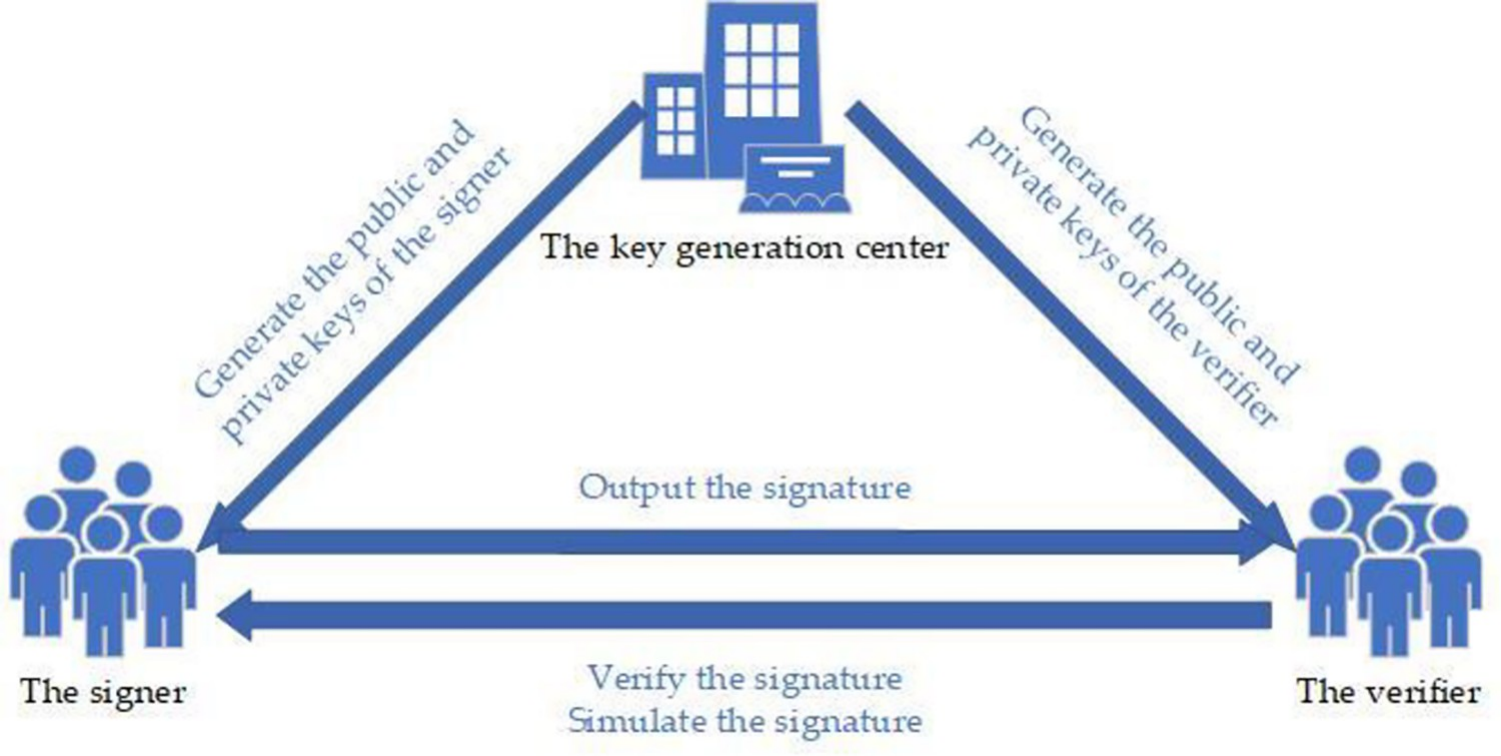
Workflow of an attribute-based strong designated verifier signature scheme.

Attribute-based signature (ABS) has strong anonymity, i.e., the verifier can only know from the signature that the attributes of the signer satisfy the access structure but not the specific information of the signer, which can effectively achieve fine-grained access control. It is of great theoretical and practical importance to study attribute-based strong designated verifier signatures by combining attribute-based signatures with strong designated verifier signatures. Workflow of an attribute-based strong designated verifier signature scheme is shown in Fig 1. There are two forms of ABS: key policy attribute-based signature (KP-ABS) and ciphertext policy attribute-based signature (CP-ABS).

### 1.1. The research problem

The currently existing attribute-based strong designated verifier signature schemes involves bilinear pairing operations in the construction process. The computational overhead of one bilinear pairing operation is approximately two to three times that of one scalar multiplication operation on the same elliptic curve [3]. Therefore, minimizing the number of calculations of bilinear pairings in the algorithm or cleverly using other operations to achieve the same algorithmic function can improve the efficiency of the attribute-base signature algorithm to some extent. In addition, the access structures of existing attribute-based strong designated verifier signatures are “And” gate or access tree structures, which have many limitations in policy expression and also affect the efficiency of attribute-based strong designated verifier signature schemes.

This study adopts the access structure of LSSS (Linear Secret Sharing Schemes) with stronger access policy expression. The linear secret sharing schemes are more efficient than the access structures “And” gates or access trees by using the linear secret reconfigurable nature of the secret to reconstruct the secret without recursive operations. Meanwhile, the new scheme is based on the elliptic curve cryptography. The scalar multiplication on elliptic curves, which is computationally lighter, is used instead of the bilinear pairing operation, which is computationally more expensive in traditional attribute-based signature schemes. The computational overhead of signing and verification in the system is reduced and the efficiency of the system is improved. This makes the computational efficiency of the proposed scheme meet the needs of resource-constrained cloud end-users.

### 1.2. Our contribution

In this paper, we propose an efficient attribute-based strong designated verifier signature scheme based on elliptic curve cryptography, and optimize the security model for an attribute-based strong designated verifier signature scheme. The security of the efficient attribute-based strong designated verifier signature scheme based on elliptic curve cryptography is analyzed. The advantages of this study are as follows.

1. To reduce the computational overhead of the system, the scheme is based on the elliptic curve cryptography, using the more lightweight scalar multiplication on the elliptic curve instead of the complex bilinear pairing operation, which effectively improves the signature and verification efficiency. The security of the scheme relies on the difficulty of the elliptic curve discrete logarithm problem (ECDLP). To the best of our knowledge, our scheme is the first attribute-based strong designated verifier signature scheme constructed using the elliptic curve cryptography.
2. The traditional “And” gate or tree access structure is less expressive, and too many redundant attributes increase the length of the ciphertext key. In order to reduce the system storage overhead, enrich the expressiveness of the access structure and save the communication overhead, we use the more expressive and computationally efficient LSSS (Linear Secret Sharing Schemes) access structure.
3. The new scheme uses a concatenated summation algorithm in the signature generation process, so that the length of the generated signature is independent of the number of attributes of the signer and does not vary with the number of attributes of the signer.

### 1.3. Organization

The remainder of this paper is organized as follows. In Section 2, we introduce some related work. In Section 3, we introduce the necessary preliminaries and provide the general form of the attribute-based strong designated verifier signature and its security model. In Section 4, we present an efficient attribute-based strong designated verifier signature scheme based on elliptic curve cryptography. In Section 5, the efficiency of the proposed scheme is analyzed. In Section 6 we summarize the full text.

## 2. Related work

In privacy-protected cloud computing environments, e-commerce, social networks, e-voting and other web application scenarios, there exists a security requirement that the signer does not want the authenticity of his digital signature to spread arbitrarily among some unauthorized users. In response to this situation, Jakobsson et al. [1] first introduced the concept of designated verifier signatures in 1996 to make the authenticity of signatures more private. In addition, considering the case that a third party can intercept the signature before it reaches the designated verifier, a strong designated verifier signature system with stronger security is proposed in the appendix of [1]. In a strong designated verifier signature scheme, the verifier must use his or her own private key to perform the verification algorithm. In this way, even if the designated verifier signature is intercepted in advance, the third party still has no signature verification capability. In 2003, Saeednia, Kremer and Markowitch [2] gave a formal definition of strong designated verifier signature and gave the first scheme for strong designated verifier signature. This scheme [2] used the Schnorr [4] signature scheme and Zheng [5] signature encryption scheme to propose a strong designated verifier signature scheme, which achieves signer identity privacy by avoiding the use of encryption algorithms and further improves the efficiency of signing and verification. A secure and flexible access control scheme and protocol for M-services based on role based access control (RBAC) [6] in the same year. In 2004, Laguillaumie et al. [7] provided the first formal description of the concept of designated verifier signatures and a formal definition of the signer identity privacy property in strong designated verifier signatures. They also improved the designated verifier signature scheme proposed by Steinfeld et al. [8] at Asiacrypt’03 using bilinear pairs and proposed a new signature scheme that possesses lower computational consumption and proved that the scheme can guarantee the privacy of the signer’s identity. Susilo et al. studied strong designated verifier signatures in the context of identity-based cryptosystems and proposed a strong designated verifier signature scheme for IBC [9], which integrates identity-based cryptosystem with strong designated verifier signature to solve the public key certificate management problem. In 2006, a usage control model to protect services and devices in ubiquitous computing environments [10], which allows the access restrictions directly on services and object documents was presented. In 2008, Zhang et al. [11] applied identity cryptography to propose a novel strong designated verifier signature scheme and proved its security to be close to the Bilinear Diffie-Hellman (BDH) hard problem under the random oracle model. Huang et al. [12] proposed a short strong designated verifier signature scheme and one of its identity-based morphing schemes, also noting that this signature is shorter than the signature lengths of all existing schemes, and finally discussing the short strong designated verifier signature under the standard model. In 2009, Kang et al. [13] demonstrated authorization attacks on some existing identity-based strong designated verifiers and proposed new signature schemes that can withstand authorization attacks. A model for privacy preserving access control which is based on variety of purposes [14] was presented in the same year. Yang et al. [15] similarly proposed a certificate-free strong designated verifier signature regime at the International Conference on Intelligence and Security in that year. In 2011, Huang et al. [16] proposed two efficient strong designated verifier signature schemes, the first one is a strong designated verifier signature scheme under the standard model and the second one is a non-authorized strong designated verifier signature scheme, and suggested that the non-authorized designated verifier signature under the standard model is still a difficult problem. Islam et al. [17] constructed provably secure certificate-free strong designated verifier signature regime using elliptic curve bilinear pairs in 2013. In 2014, Wang et al. proposed a strong designated verifier signature scheme that is recognizable by the signer [18]. In [18], if permission is offered, the signatory can acknowledge that the signature is his own. In 2015, Jiang et al. proposed an identity-based online and offline designated verifier signature scheme [19]. In 2015, Zhang proposed a strong designated verifier signature scheme that resists replay attacks [20]. In 2017, Masoumeh et al. [21] proposed a strong designated verifier signature scheme. Ge et al. [22] proposed two SDVS schemes that guarantee the privacy of the signer. In 2019, Han et al. [23] proposed certificateless SDVS, which satisfies the requirements of verifiability, non-authorizability, non-transmissibility, and signer ambiguity. In 2020, Zhang et al. [24] proposed secure and efficient quantum DVS scheme, which is theoretically secure and a distributed memetic algorithm (DMA) is proposed for enhancing database privacy and utility [25]. In 2022, Venkateswaran et al. proposed the use of a neuro Deep learning wireless intrusion detection system that distinguishes the attacks in MANETs [26]. Dharmaraj et al. proposed a feature selection and majority voting based solutions for detecting intrusions [27]. In the same year, a novel three-layer DDE framework with adaptive resource allocation (DDE-ARA) [28] was proposed and a multitasking database fragmentation problem for privacy preservation requirements [29] is formally defined. During the same period, Yin et al. [30] proposed a modality-aware graph convolutional network (MAGCN) module to embed multimodality entity attributes and topological graph connectivity features into a unified lower dimensional feature space to boost link prediction performance. In 2023, Ravinder et al. [31] proposed a proactive approach based on natural language processing and deep learning that can enable online platforms to actively look for the signs of antisocial behaviour and intervene before it gets out of control. Ge et al. proposed a distributed prediction-randomness framework for the evolutionary dynamic multiobjective partitioning optimization of databases [32] and a distributed cooperative coevolutionary genetic algorithm (DCCGA) to optimize the MODP problem [33].

In 2008, Maji [34] et al. first proposed the attribute-based signature (ABS) scheme based on the IBS scheme. The access structure consists of a threshold structure consisting of "And" and "Or" and finally proves its security under a general group model. In 2010 Li et al. [35] et al. proposed three schemes, the first one is an ABS scheme with threshold predicates, the second one is an ABS scheme without random oracle machines, and the third one is an ABS scheme with multi-attribute authorities. In 2011, Maji et al. [36] presented a general framework for constructing attribute-based signatures and gave several concrete examples using bilinear pairs. In 2012, Sun et al. [37] proposed a threshold attribute-based signature scheme without trusted central attribute authority that is not only unforgeable under selective attribute and adaptive selective plaintext attacks, but is also resistant to conspiracy attacks. In 2013, Ma et al. [38] designed a secure and provable threshold-based attribute signature scheme. 2014, Tang et al. [39] constructed an ABS scheme with limited circuit depth using a mathematical tool of multilinear mapping, further enriching the predicate expression capability. In 2015, Nandi et al. [40] constructed an ABS scheme supporting multiple access methods with control methods including Boolean formulas and conventional languages. In 2016, Sakai et al. [41] proposed an ABS scheme supporting arbitrary circuit depth with good expressiveness via bilinear pairs. In 2017, for application to electronic medical record systems and to reduce the computational overhead, Moro et al. [42] proposed an ABS scheme that can support a tree access structure. Su et al. [43] proposed an attribute-based signature scheme with attribute revocation to protect the privacy of the user’s identity. Lu et al. [44] propose an efficient traceable constant-size attribute-based ring signature scheme for electronic health record system, focusing on fine-grained authentication and traceability of file publishers. Ma et al. [45] present an attribute-based blind signature scheme based on elliptic curve cryptography (ECC), and the security of new scheme is proved under the difficulty of the elliptic curve discrete logarithm problem (ECDLP). The access structure of the scheme uses the LSSS matrix. In the same year, an efficient pairing-free attribute-based blind signature scheme based on ordered binary decision diagram [46] is proposed.

Currently, the following works are available on attribute-based strong designated verifier signature. In 2009 and 2012, Shao et al. [47] and Fan et al. [48] proposed attribute-based strong designated verifier signature schemes, respectively, but these two schemes have multiple bilinear pairing operations in the signature and verification process, which makes the overall efficiency of the schemes inefficient. The attribute-based designated verifier signature scheme proposed by Tang et al. [49] in 2014 and the deniable attribute-based designated confirmer signature scheme proposed by Ren et al. [50] in the same year are not true attribute signatures because the secret value *y*_1_ is incorrectly given to the signer in the public-private key extraction algorithm. Based on the paper [50], in 2016, Yan Ren [51] proposed a deniable attribute-based designated confirmer signature scheme under no random prediction model. In 2020 Zhang et al. [52] used a key strategy and then proposed an attribute-based designation confirmer signature scheme with a monotonic Boolean circuit for the access structure using multilinear mapping. The access structure of both schemes is a “And” gate or access tree structure, which involves bilinear pairing operations in the signature and verification process, making the overall system inefficient.

Most existing attribute-based strong designated verifier signature schemes involve complex bilinear pairing operations in the construction process, which are considered to be the most computationally expensive operations in pairing-based cryptographic protocols [53]. This makes these solutions overall inefficient and difficult to apply to cloud terminal scenarios or resource-constrained devices. Therefore, minimizing the number of calculations of bilinear pairings in the algorithm or cleverly using other operations to achieve the same algorithmic function can improve the algorithmic efficiency of the attribute-based strong designated verifier signature scheme to some extent. The design of the access structure is also a fairly important part of the construction of an attribute-based strong designated verifier signature scheme. A better access structure not only improves the efficiency of the system and the expressiveness of the access policy, but also reduces the number of attributes that need to be embedded in the signature to shorten the signature length and reduce the communication and storage overhead.

## 3. Preliminaries

In this section, we introduce linear secret sharing schemes (LSSS), elliptic curve cryptography, and the necessary security assumption. In addition, the definition and security model of the attribute-based strong designated verifier signature algorithm are provided.

### 3.1. Linear secret sharing schemes

*Definition 1 (Access Structure)* [54]. Suppose that the set of *n* participants is {*P*_1_,*P*_2_,⋯,*P*_*n*_}, and $P=2^{\left\{P_{1},P_{2},\ldots ,P_{n}\right\}}$. If the set *A* is a non-empty subset of the set {*P*_1_,*P*_2_,⋯,*P*_*n*_}, then it satisfies *A*⊆*P*\{*Φ*}. If ∀*B*,*C*, satisfying *B*∈*A* and *B*⊆*C*, has *C*∈*A*, then *A* is said to be a monotonic access structure.

*Definition 2 (Linear Secret Sharing Schemes Access Structure)*. Let {*P*_1_,*P*_2_,⋯,*P*_*n*_} be the set of a series of participants, let *M*_*T*_ be the matrix of *s*×*t*, and *ρ*:{1,2,⋯,*s*}→*P* be the mapping of each row of the matrix to one of the participants in the set. According to the definition of a linear secret sharing scheme [54], linear secret sharing schemes access structure is defined as the following two algorithms.

1.*Distribute*(*M*_*T*_,*ρ*,*α*). The input matrix *M*_*T*_ with row *s* and column *t*, the mapping function *ρ* and the secret value *α*∈Z_p_*, randomly selected $\alpha_{2},\ldots ,\alpha_{t}\in {Z_{p}}^{*}$, forms the vector *v* =(*α*,*α*_2_,⋯,*α*_*t*_), and then output the *s* shared values ${\left\{\lambda_{i}=M_{T_{i}}\cdot v\right\}}_{i\in [1,s]}$ of attribute *ρ*(*i*), where $M_{T_{i}}$ is the *i*-th row vector of matrix *M*_*T*_.

2.*Reconstruct*(*M*_*T*_,*ρ*,*W*). The input matrix *M*_*T*_ with row *s* and column *t*, the mapping function *ρ* and the set of authorized attributes *W*∈*P*. According to the Gaussian elimination method, the set of reconstruction constants ${\left\{w_{i}\right\}}_{i\in I}$ can be found in polynomial time, satisfying $\sum_{i\in I}M_{T_{i}}w_{i}=(1,0,\ldots ,0)$, i.e., $\sum_{i\in I}\lambda_{i}w_{i}=\alpha$. Then output ${\left\{w_{i}\right\}}_{i\in I}$, where *I* = {*i*∈[1,*s*]:*ρ*(*i*)∈*W*}.

### 3.2. ECDLP

In the mid-1980s, Koblitz [55] and Miller [56] respectively proposed the elliptic curve cryptography (ECC), whose security relies on the intractability of the discrete logarithm problem (ECDLP) on the elliptic curve group. The elliptic curve discrete logarithm problem can be described as follows:

Let *F*_*p*_ denote a finite field and *E* be an elliptic curve over *F*_*p*_. The point *G* as the base point of this elliptic curve *E*(*F*_*P*_), *n* is the order of *G*. A point *Q*∈*E*(*F*_*P*_), The elliptic curve discrete logarithm problem (ECDLP) is the search for an integer *l*∈[0,*n*−1] such that *Q* = *lG*. For any algorithm B, the probability of solving the ECDLP is defined as follows,

$$Adv^{ECDLP}(B)=\mathrm{Pr}\left[B(G,lG)=l,\;l\in [0,n]\right]$$

*Definition 3*. The elliptic curve discrete logarithm problem (ECDLP) is said to be a hard problem if the probability of any algorithm B solving the ECDLP is negligible.

### 3.3. A generic definitions of an attribute-based strong designated verifier signature scheme

An attribute-based strong designated verifier signature scheme generally includes the following five algorithms.

#### 3.3.1. Setup


$$Setup(k)\to \{P_{pub},MSK,params\}$$

A probabilistic algorithm has as input a security parameter *k*, outputs a system master key *MSK* and a master public key *P*_*pub*_, and a system public parameter *params*.

#### 3.3.2. Extract


$$Extract(params,MSK,T_{A},T_{B})\to \{SK_{A},PK_{A},SK_{B},PK_{B}\}$$

A probabilistic algorithm with input system parameters *params*, master key *MSK*,access structure *T*_*A*_, and output private key *SK*_*A*_ and public key *PK*_*A*_ of the signer. Similarly, input system parameters *params*, master key *MSK*, access structure *T*_*B*_, and output private key *SK*_*B*_ and public key *PK*_*B*_ of the signer.

#### 3.3.3. Sign


$$Sign(M,params,SK_{A},PK_{A},PK_{B})\to \sigma$$

A probabilistic algorithm with input system parameters *params*, private key *SK*_*A*_ and public key *PK*_*A*_ of the signer, public key *PK*_*B*_ of the verifier, message *M*, output the signature *σ* of message *M*.

#### 3.3.4. Verify


$$Verify(params,PK_{A},PK_{B},M,\sigma )\to \{1,0\}$$

A deterministic algorithm with input system parameters *params*, the public key *PK*_*A*_ of the signer, the private key *SK*_*B*_ of the verifier, the message *M* and its signature *σ*, and output whether the signature verification passes or not. If the signature *σ* is valid, output 1, otherwise output 0.

#### 3.3.5. Simulate


$$Simulate(M,\sigma ,PK_{A},SK_{B},params)\to \sigma^{\prime }$$

A deterministic algorithm that takes as input the message *M* and its signature *σ*, the public key *PK*_*A*_ of the signer, the private key *SK*_*B*_ of the designated verifier and other public parameters *params*, and outputs a simulated signature *σ*′ of the message *M*.

### 3.4. A security model of an attribute-based strong designated verifier signature scheme

A secure attribute-based strong designated verifier signature scheme needs to satisfy correctness, signer identity anonymity, unforgeability, and privacy non-transmissibility.

#### 3.4.1. Correctness

An attribute-based strong designated verifier signature scheme

π=(Setup,Extract,Sign,Verify,Simulate)

assuming that the set *W*_*A*_ of attributes owned by the signer satisfies the access structure *T*_*A*_, the signer outputs the designated verifier signature *σ* = *Sign*(*M*,*params*,*SK*_*A*_,*PK*_*A*_,*PK*_*B*_) for message *M*. Assuming that the set *W*_*B*_ of attributes owned by the designated verifier satisfies the access structure *T*_*B*_, the designated verifier signature *σ* generated by the signer must be verified by the verifier, there must be

$$Verify(params,PK_{A},PK_{B},M,\sigma )=1$$

#### 3.4.2. Signer identity anonymity

An attribute-based strong designated verifier signature scheme

π=Setup,Extract,Sign,Verify,Simulate)

signer identity anonymity without access to the private key of the signer or the designated verifier can be defined as a series of games between adversary and challenger as follows.

1. *Init*: Adversary A selects the attribute set ${W_{A}}^{*}$ and the set ${W_{B}}^{*}$ of attributes owned by the designated verifier to be challenged, and sends them to challenger C.
2. *Setup*: Challenger C chooses security parameters *k*, computes (*params*,*MSK*)←*Setup*(*k*), and sends public parameters *params* to adversary A.
3. *Queries*: Adversary A is allowed to perform polynomial subadaptive queries.
  - Key extraction queries. Adversary A sends the LSSS access structure *T*_*A*_ and *T*_*B*_ to challenger C, if $T_{A}({W_{A}}^{*})\neq 1$ and $T_{B}({W_{B}}^{*})\neq 1$, challenger C runs algorithm *Extract* and returns to adversary A the public-private key (*PK*_*A*_,*SK*_*A*_) of the signer and the public-private key (*PK*_*B*_,*SK*_*B*_) of the verifier.
  - Signature queries. Adversary A sends the attribute set *W*_*A*_, the message *M*, and the attribute set of the verifier *W*_*B*_ to challenger C. Challenger C invokes the *Sign* algorithm to generate signature *σ*, which is sent to adversary A.
  - Verify queries. Adversary A sends message *M* and signature *σ* to challenger C, requesting challenger C to verify that signature *σ* is signed by a signer with attribute *W*_*A*_ and designated verifier attribute *W*_*B*_. If *σ* is a signature generated by a legitimate signer with attribute set *W*_*A*_, then challenger C returns "1", if not, then returns "0".
4. *Challenge*: Adversary A submits two plaintexts of equal length *M*_0_ and *M*_1_,to challenger C, the attribute set *W*_*A*_ of the signer and the attribute set *W*_*B*_ of the verifier. Challenger C performs a random coin flip, set to *b*∈{0,1}, and generates a strong designated verifier signature $\sigma_{b}=Sign({W_{A}}^{*},{W_{B}}^{*},M_{b})$, which is sent to adversary A.
5. *Guess*: Adversary A outputs a guess *b*′ for *b*. Before giving the guess, adversary A can make signature queries to challenger C other than *M*_0_ and *M*_1_ and verify queries other than *σ*_*b*_. If *b*′ = *b*, output "1", otherwise, output "0".

We define *Adv*^*anony*^(1^*λ*^) to be the advantage over 1/2 of A in the above game.

*Definition 4 (Signer identity anonymity)*. An attribute-based strong designated verifier signature scheme satisfies signer identity anonymity under a choice message attack if there exists no adversary A can win the above game with non-negligible advantage *Adv*^*anony*^(1^*λ*^).

#### 3.4.3. Unforgeability

An attribute-based strong designated verifier signature scheme

π=(Setup,Extract,Sign,Verify,Simulate)

it is computationally infeasible to construct a legitimate attribute-based strong designated verifier signature scheme without obtaining the signer’s or designated verifier’s private key. Unforgeability under selective attribute set and selective messages attack can be defined as the following game in polynomial time between adversary A and challenger C.

1. *Init*: Adversary A selects the attribute set ${W_{A}}^{*}$ and the set ${W_{B}}^{*}$ of attributes owned by the designated verifier to be challenged, and sends them to challenger C.
2. *Setup*: Challenger C chooses security parameters *k*, computes (*params*,*MSK*)←*Setup*(*k*), and sends public parameters *params* to adversary A.
3. *Queries*: Adversary A is allowed to perform polynomial subadaptive queries.
  - Key extraction queries. Adversary A sends the LSSS access structure *T*_*A*_ and *T*_*B*_ to challenger C, if $T_{A}({W_{A}}^{*})\neq 1$ and $T_{B}({W_{B}}^{*})\neq 1$, challenger C runs algorithm *Extract* and returns to adversary A the public-private key (*PK*_*A*_,*SK*_*A*_) of the signer and the public-private key (*PK*_*B*_,*SK*_*B*_) of the verifier.
  - Signature queries. Adversary A sends the attribute set *W*_*A*_, the message *M*, and the attribute set of the verifier *W*_*B*_ to challenger C. Challenger C invokes the *Sign* algorithm to generate signature *σ*, which is sent to adversary A.
  - Verify queries. Adversary A sends message *M* and signature *σ* to challenger C, requesting challenger C to verify that signature *σ* is signed by a signer with attribute *W*_*A*_ and designated verifier attribute *W*_*B*_. If *σ* is a signature generated by a legitimate signer with attribute set *W*_*A*_, then challenger C returns "1", if not, then returns "0".
4. *Forgery*: Adversary A outputs the signature σ*of message M* with the corresponding signer’s attribute set ${W_{A}}^{*}$, the corresponding verifier’s attribute set ${W_{B}}^{*}$, and the following three conditions are satisfied
$$Verify(params,{W_{A}}^{*},M^{*},\sigma^{*},SK_{B})=1.$$ (1)

Adversary A did not conduct a signature queries $\left(M^{*},{W_{A}}^{*},{W_{B}}^{*}\right)$.

The access structures *T*_*A*_ and *T*_*B*_ for conducting either query, both satisfy $T_{A}({W_{A}}^{*})\neq 1$ and $T_{B}({W_{B}}^{*})\neq 1$.

*Definition 5 (Unforgeability)*. An attribute-based strong designated verifier signature scheme is unforgeable under the selective attributes and selective messages attacks when the probability that adversary A can successfully win the above game in polynomial time is negligible.

#### 3.4.4. Privacy non-transmissibility

An attribute-based strong designated verifier signature scheme

π=(Setup,Extract,Sign,Verify,Simulate)

satisfies privacy non-transmissibility means that given a message *M* and a strong designated verifier signature *σ*, the probability that a third party can determine in polynomial time whether the signature *σ* was generated by the signer or the verifier is negligible. Privacy non-transmissibility can be defined as the following game in polynomial time between adversary A and challenger C.

1. *Init*: Adversary A selects the attribute set ${W_{A}}^{*}$ and the set ${W_{B}}^{*}$ of attributes owned by the designated verifier to be challenged, and sends them to challenger C.
2. *Setup*: Challenger C chooses security parameters *k*, computes (*params*,*MSK*)←*Setup*(*k*), and sends public parameters *params* to adversary A.
3. *Queries*: Adversary A is allowed to perform polynomial subadaptive queries.
  - Key extraction queries. Adversary A sends the LSSS access structure *T*_*A*_ and *T*_*B*_ to challenger C, if $T_{A}({W_{A}}^{*})\neq 1$ and $T_{B}({W_{B}}^{*})\neq 1$, challenger C runs algorithm *Extract* and returns to adversary A the public-private key (*PK*_*A*_,*SK*_*A*_) of the signer and the public-private key (*PK*_*B*_,*SK*_*B*_) of the verifier.
  - Signature queries. Adversary A sends the attribute set *W*_*A*_, the message *M*, and the attribute set of the verifier *W*_*B*_ to challenger C. Challenger C invokes the *Sign* algorithm to generate signature *σ*, which is sent to adversary A.
  - Verify queries. Adversary A sends message *M* and signature *σ* to challenger C, requesting challenger C to verify that signature *σ* is signed by a signer with attribute *W*_*A*_ and designated verifier attribute *W*_*B*_. If *σ* is a signature generated by a legitimate signer with attribute set *W*_*A*_, then challenger C returns "1", if not, then returns "0".
4. *Challenge*: Challenger C runs *Simulate* algorithm and generates a signature *σ*′, which is sent to challenger A. The signature verification equation still holds. If adversary A can distinguish between the signature *σ* generated by the signer and signature *σ*′ generated by the challenger C in polynomial time, then adversary A wins.

*Definition 6 (Privacy non-transmissibility)*. An attribute-based strong designated verifier signature scheme satisfies privacy non-transmissibility when the probability that adversary A can successfully win the above game in polynomial time is negligible.

**Fig 2:**
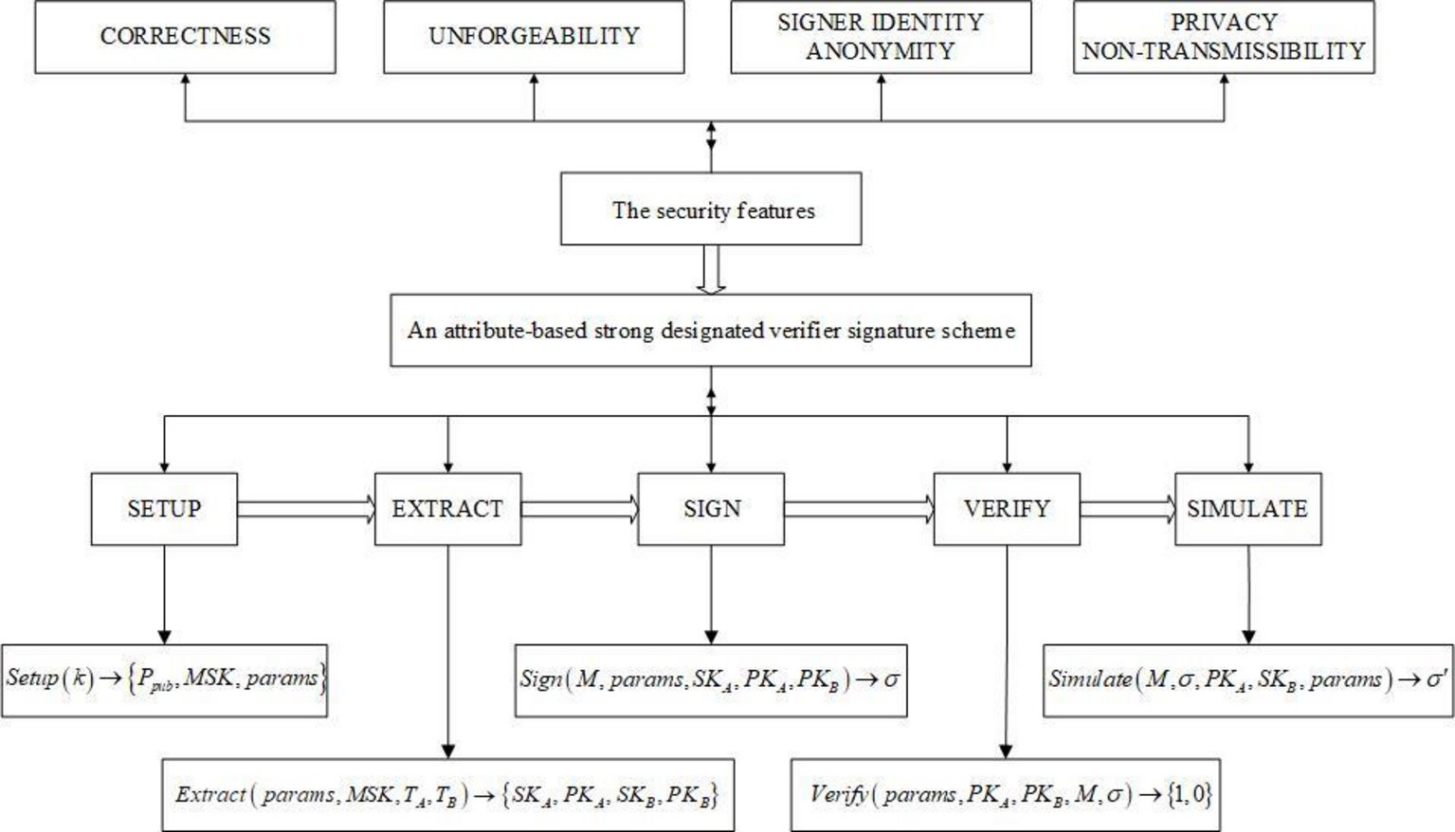
System model of an attribute-based strong designated verifier signature scheme.

System model of an attribute-based strong designated verifier signature scheme is shown in Fig 2.

## 4. An efficient attribute-based strong designated verifier signature scheme based on elliptic curve cryptography

Most of the existing attribute-based strong designated verifier signature schemes involve complex bilinear pairing operations and the “And” gate or access tree access structure used in the scheme construction has many limitations in policy expression, which makes the signing and verification process computationally inefficient. To address this issue, an efficient attribute-based strong designated verifier signature scheme based on elliptic curve cryptography is proposed and its security is analyzed in this section.

### 4.1. Our construction

In this section we propose an efficient attribute-based strong designated verifier signature scheme based on elliptic curve cryptography including the following five algorithms.

#### 4.1.1. Setup

The finite field *GF*(*p*) of order *p* is chosen, *E* is an elliptic curve defined on *GF*(*p*), and the system chooses the point *G* as the base point of this elliptic curve. Assume that the set of attributes in the system is *U* = {1,2,⋯,*n*} and *i* is one of the attributes. $H:{\left\{0,1\right\}}^{*}\to {Z_{p}}^{*}$ is a cryptographically secure hash function. Randomly select $\alpha \in {Z_{p}}^{*}$ and compute *P*_*pub*_ = *αG*. For each attribute *i*∈*U*, randomly select the secret value $z_{i}\in {Z_{p}}^{*}$ and compute *h*_*i*_ = *z*_*i*_*G*.

Output public parameters *params* = {*p*,*G*,*H*,*h*_1_,*h*_2_,⋯,*h*_*n*_,*P*_*pub*_}. The master key is *MSK* = {*α*,*z*_1_,*z*_2_,⋯,*Z*_*n*_}.

#### 4.1.2. Extract

Assume that the access structure *T*_*A*_ of the signer is (*L*_*A*_,*ρ*_*A*_), *L*_*A*_ is a matrix of rows and columns *S*_*A*_ and *t*_*A*_. The function *ρ*_*A*_ is a mapping of rows to attributes about *L*_*A*_. Each row $L_{A_{j}}$ of *L*_*A*_ corresponds to an attribute $\rho_{A_{j}}$.

The key generation center randomly selects $r_{A_{2}},r_{A_{3}},\ldots ,r_{A_{t}}\in {Z_{p}}^{*}$, constructs the vector ${\vec{v}}_{A}=(\alpha ,r_{A_{2}},r_{A_{3}},\ldots ,r_{A_{t}})$, calculates the secret value $\lambda_{A_{i}}={\vec{L}}_{A_{i}}\cdot {\vec{v}}_{A}$ for each row *i*∈[1,*S*_*A*_] of the LSSS matrix *L*_*A*_, and then calculates $d_{A_{i}}=\lambda_{A_{i}}+z_{\rho_{A}(i)}$.

Output the private key of the signer as $SK_{A}={\left\{d_{A_{i}}\right\}}_{i\in [1,s_{A}]}$. Compute $D_{A_{i}}=d_{A_{i}}G$ and the public key of the signer as $PK_{A}={\left\{D_{A_{i}}\right\}}_{i\in [1,s_{A}]}$.

Assume that the access structure *T*_*B*_ of the verifier is (*L*_*B*_,*ρ*_*B*_), *L*_*B*_ is a matrix of rows and columns *S*_*B*_ and *t*_*B*_. The function *ρ*_*B*_ is a mapping of rows to attributes about *L*_*B*_. Each row $L_{B_{j}}$ of *L*_*B*_ corresponds to an attribute $\rho_{B_{j}}$.

The key generation center randomly selects $r_{B_{2}},r_{B_{3}},\ldots ,r_{B_{t}}\in {Z_{p}}^{*}$, constructs the vector ${\vec{v}}_{B}=(\alpha ,r_{B_{2}},r_{B_{3}},\ldots ,r_{B_{t_{B}}})$, calculates the secret value $\lambda_{B_{i}}={\vec{L}}_{B_{i}}\cdot {\vec{v}}_{B}$ for each row *i*∈[1,*S*_*B*_] of the LSSS matrix *L*_*B*_, and then calculates $d_{B_{i}}=\lambda_{B_{i}}+z_{\rho_{B}(i)}$.

Output the private key of the signer as $SK_{B}={\left\{d_{B_{i}}\right\}}_{i\in [1,s_{B}]}$. Compute $D_{A_{i}}=d_{B_{i}}G$ and the public key of the signer as $PK_{B}={\left\{D_{B_{i}}\right\}}_{i\in [1,s_{B}]}$.

#### 4.1.3. Sign

If the attribute set *W*_*A*_ of the signer satisfies the access structure *T*_*A*_, it must be possible to find a set of constant $\left\{\omega_{A_{i}}\in {Z_{p}}^{*},i\in \varpi_{A}\right\}$ in polynomial time such that $\sum_{i\in \varpi_{A}}\omega_{A_{i}}L_{A_{i}}=(1,0,\ldots ,0)$, where $\varpi_{A}=\{i\in [1,s_{A}]:\rho_{A}(i)\in W_{A}\}$.

The signer signs the message as follows.

Randomly select $e\in {Z_{p}}^{*}$ and compute *R* = *eM*, *r* = *H*(*R*). Randomly select $k_{1}\in {Z_{p}}^{*}$ and compute $s=k_{1}-r\sum_{i\in \varpi_{A}}d_{A_{i}}\omega_{A_{i}}$.

If the attribute set *W*_*B*_ of the verifier satisfies the access structure *T*_*B*_, it must be possible to find a set of constant $\left\{\omega_{B_{i}}\in {Z_{p}}^{*},i\in \varpi_{B}\right\}$ in polynomial time such that $\sum_{i\in \varpi_{B}}\omega_{B_{i}}L_{B_{i}}=(1,0,\ldots ,0)$, where $\varpi_{B}=\{i\in [1,s_{B}]:\rho_{B}(i)\in W_{B}\}$.

The signer picks $k_{2}\in {Z_{p}}^{*}$ randomly and computes $V=k_{2}\cdot \sum_{i\in \varpi_{B}}D_{B_{i}}$ and $Z=k_{1}\sum_{i\in \varpi_{B}}D_{B_{i}}+k_{2}G$.

The signer sends the signature *σ* = (*R*,*s*,*V*,*Z*) to the verifier.

#### 4.1.4. Verify

After the verifier receives the signature *σ*, calculate *r* = *H*(*R*).

Verify that the equation $Z=\sum_{i\in \varpi_{B}}d_{B_{i}}[sG+r(P_{pub}+\sum_{i\in \varpi_{A}}h_{A_{i}}\omega_{A_{i}})]+{\left(\sum_{i\in \varpi_{B}}d_{B_{i}}\right)}^{-1}V$ Whether it holds. If it holds, accept the signature, if not, reject it.

#### 4.1.5. Simulate

The verifier randomly selects $k\in {Z_{p}}^{*}$ and computes $Z=k\sum_{i\in \varpi_{B}}D_{B_{i}}$.

The verifier computes *r* = *H*(*R*), *s* = *k*, $V=-r{\left(\sum_{i\in \varpi_{B}}d_{B_{i}}\right)}^{2}(P_{pub}+\sum_{i\in \varpi_{A}}h_{A_{i}}\omega_{A_{i}})$, generating a simulated signature *σ*′ = (*R*,*s*,*V*,*Z*) such that the verification equation

$$Z=\sum_{i\in \varpi_{B}}d_{B_{i}}[sG+r(P_{pub}+\sum_{i\in \varpi_{A}}h_{A_{i}}\omega_{A_{i}})]+{\left(\sum_{i\in \varpi_{B}}d_{B_{i}}\right)}^{-1}V$$ (2)

also holds.

### 4.2. Security analysis

In this section we analyze the security of an efficient attribute-based strong designated verifier signature scheme based on elliptic curve cryptography. The security features mainly include correctness, signer identity anonymity, unforgeability, and privacy non-transmissibility.

#### 4.2.1. Correctness

The efficient attribute-based strong designated verifier signature scheme based on elliptic curve cryptography proposed by us satisfies the correctness.

*Proof*: When the attribute set *W*_*A*_ of the signer satisfies the access structure *T*_*A*_, the same set of reconstruction constants $\left\{\omega_{A_{i}}\in {Z_{p}}^{*},i\in \varpi_{A}\right\}$ as the signer can be found, where $\varpi_{A}=\{i\in [1,s_{A}]:\rho_{A}(i)\in W_{A}\}$. According to the properties of the LSSS matrix, $\sum_{i\in \varpi_{A}}\lambda_{A_{i}}\omega_{A_{i}}=\alpha$ and the system parameters *params*, the correctness of the signature *σ* is verified as follows.


$$Z=k_{1}\sum_{i\in \varpi_{B}}D_{B_{i}}+k_{2}G$$

$$=k_{1}\sum_{i\in \varpi_{B}}d_{B_{i}}G+k_{2}G$$

$$=(s+r\sum_{i\in \varpi_{A}}d_{A_{i}}\omega_{A_{i}})\sum_{i\in \varpi_{B}}d_{B_{i}}G+k_{2}G$$

$$=s\sum_{i\in \varpi_{B}}d_{B_{i}}G+r\sum_{i\in \varpi_{A}}d_{A_{i}}\omega_{A_{i}}\sum_{i\in \varpi_{B}}d_{B_{i}}G+k_{2}G$$

$$=\sum_{i\in \varpi_{B}}d_{B_{i}}(sG+r\sum_{i\in \varpi_{A}}d_{A_{i}}\omega_{A_{i}}G)+k_{2}G$$

$$=\sum_{i\in \varpi_{B}}d_{B_{i}}[sG+r\sum_{i\in \varpi_{A}}(\lambda_{A_{i}}+z_{\rho_{A}(i)})\omega_{A_{i}}G]+k_{2}G$$

$$=\sum_{i\in \varpi_{B}}d_{B_{i}}[sG+r(P_{pub}+\sum_{i\in \varpi_{A}}h_{A_{i}}\omega_{A_{i}})]+{\left(\sum_{i\in \varpi_{B}}d_{B_{i}}\right)}^{-1}V$$

#### 4.2.2. Signer identity anonymity

If the probability that an adversary A can distinguish a legitimate attribute-based strong designated verifier signature in polynomial time without obtaining a signer or designated verifier private key is no greater than 1/2, the efficient attribute-based strong designated verifier signature scheme based on elliptic curve cryptography proposed by us satisfies the signer identity anonymity.

*Proof*: The game between adversary A and challenger C is as follows.

1. *Init*. Adversary A selects the attribute set ${W_{A}}^{*}$ of the signer and the attribute set ${W_{B}}^{*}$ owned by the designated verifier to be challenged, and sends them to challenger C.
2. *Setup*. challenger C selects a security parameter *k* and simulates the generation of public parameters as follows.
  Randomly selects $x\in {Z_{p}}^{*}$, calculate *P*_*pub*_ =*xG*. For each attribute *i*∈*U*, randomly select the secret value $z_{i}\in {Z_{p}}^{*}$ and compute *h*_*i*_ =*z*_*i*_*G*. Let $H:{\left\{0,1\right\}}^{*}\to {Z_{p}}^{*}$ be a secure cryptographic hash function. Challenger C generates the public parameter *params* = {*p*,*G*,*H*,*h*_1_,*h*_2_,⋯,*h*_*n*_,*P*_*pub*_} and the master key *MSK* = {*x*,*z*_1_,*z*_2_,⋯,*z*_*n*_} to adversary A.
3. *Queries*. Adversary A can perform polynomial times of key extraction queries, signature queries, and verify queries to challenger C.
  - Key extraction queries. Adversary A sends the access structure *T*_*A*_ satisfying $T_{A}({W_{A}}^{*})\neq 1$ to challenger C. Challenger C randomly selects $r_{A_{1}},r_{A_{2}},\ldots ,r_{A_{t}}\in {Z_{p}}^{*}$, constructs vector ${\vec{v}}_{A}=(x,r_{A_{2}},r_{A_{3}},\ldots ,r_{A_{t}})$, computes $\lambda_{A_{i}}={\vec{L}}_{A_{i}}\cdot {\vec{v}}_{A}$ for each row *i*∈[1,*S*_*A*_] of the LSSS matrix *L*_*A*_, and then computes the private key of the signer as $d_{A_{i}}=\lambda_{A_{i}}+z_{\rho_{A}(i)}$ and the public key of the signer as $PK_{A}={\left\{D_{A_{i}}\right\}}_{i\in [1,s_{A}]}$.

Adversary A sends the access structure *T*_*B*_ satisfying $T_{B}({W_{B}}^{*})\neq 1$ to challenger C. Challenger C randomly selects $r_{B_{1}},r_{B_{2}},\ldots ,r_{B_{t}}\in {Z_{p}}^{*}$ and constructs the vector ${\vec{v}}_{B}=(x,r_{B_{2}},r_{B_{3}},\ldots ,r_{B_{t}})$. For each row *i*∈[1,*S*_*B*_] of the LSSS matrix *L*_*A*_, compute $\lambda_{B_{i}}={\vec{L}}_{B_{i}}\cdot {\vec{v}}_{B}$. Compute the private key of the verifier as $d_{B_{i}}=\lambda_{B_{i}}+z_{\rho_{B}(i)}$ and the public key of the verifier as $PK_{B}={\left\{D_{B_{i}}\right\}}_{i\in [1,s_{B}]}$.

- Signature queries. Adversary A sends the attribute set *W*_*A*_ of the signer, message *M*, and verifier attribute set *W*_*B*_ of the verifier to challenger C. Challenger C signs message *M* according to the signature step.

If the attribute set *W*_*A*_ satisfies the access structure *T*_*A*_, then one can obtain a set of constants $\left\{\omega_{A_{i}}\in {Z_{p}}^{*},i\in \varpi_{A}\right\}$ such that $\sum_{i\in \varpi_{A}}\omega_{A_{i}}L_{A_{i}}=(1,0,\ldots ,0)$, where $\varpi_{A}=\{i\in [1,s_{A}]:\rho_{A}(i)\in W_{A}\}$. Randomly select $e\in {Z_{p}}^{*}$ and calculate *R* = *eM*, *r* = *H*(*R*), randomly select $k_{1}\in {Z_{p}}^{*}$ and calculate $s=k_{1}-r\sum_{i\in \varpi_{A}}d_{A_{i}}\omega_{A_{i}}$.

If the attribute set *W*_*B*_ of the verifier satisfies the access structure *T*_*B*_, then a set of constants $\left\{\omega_{B_{i}}\in {Z_{p}}^{*},i\in \varpi_{B}\right\}$ can be found in polynomial time such that $\sum_{i\in \varpi_{B}}\omega_{B_{i}}L_{B_{i}}=(1,0,\ldots ,0)$, where $\varpi_{B}=\{i\in [1,s_{B}]:\rho_{B}(i)\in W_{B}\}$.

Challenger C picks $k_{2}\in {Z_{p}}^{*}$ randomly and computes $V=k_{2}\sum_{i\in \varpi_{B}}D_{B_{i}}$ and $Z=k_{1}\sum_{i\in \varpi_{B}}D_{B_{i}}+k_{2}G$.

Challenger C sends the signature *σ* = (*R*,*s*,*V*,*Z*) to adversary A.

- Verify queries. Adversary A sends signature *σ* = (*R*,*s*,*V*,*Z*) to challenger C. Challenger C verifies the signature according to the verification algorithm as follows.

Challenger C computes *r* = *H*(*R*) and verifies that equation

$$Z=\sum_{i\in \varpi_{B}}d_{B_{i}}[sG+r(P_{pub}+\sum_{i\in \varpi_{A}}h_{A_{i}}\omega_{A_{i}})]+{\left(\sum_{i\in \varpi_{B}}d_{B_{i}}\right)}^{-1}V.$$ (3)

If it holds, then challenger C returns "1" to adversary A. Otherwise, it returns "0".

4. *Challenge*.Adversary A submits to challenger C two plaintexts of equal length *M*_0_ and *M*_1_, the attribute set *W*_*A*_ of the signer and the attribute set *W*_*B*_ of the designated verifier. Challenger C performs a random coin flip, set to *b*∈{0,1}. Invoke signature algorithm *Sign*, randomly select $e\in {Z_{p}}^{*}$, compute *R* = *eM*_*b*_ and *r* = *H*(*R*). Randomly select $k_{1}\in {Z_{p}}^{*}$, compute $s=k_{1}-r\sum_{i\in \varpi_{A}}d_{A_{i}}\omega_{A_{i}}$. Randomly select $k_{2}\in {Z_{p}}^{*}$, compute $V=k_{2}\sum_{i\in \varpi_{B}}D_{B_{i}}$ and $Z=k_{1}\sum_{i\in \varpi_{B}}D_{B_{i}}+k_{2}G$.

Generate the designated verifier signature *σ*_b_ = *Sign*(*W*_*A*_, *W*_*B*_,*M*_*b*_) = (*R*,*s*,*V*,*Z*) to send to adversary A.

5. *Output*. Adversary A outputs a guess *b*′ for *b*. Before giving the guess, adversary A can make signature queries other than *M*_0_ and *M*_1_ and verify queries other than *σ*_*b*_ to challenger C.

In order to obtain the value of *M*_*b*_, *e* must be derived from *R* = *eM*_*b*_. Since *e* is randomly selected, the probability that adversary A determines the true value of *M*_*b*_ in polynomial time does not exceed 1/2. Therefore, the proposed scheme satisfies signer identity anonymity.

#### 4.2.3. Unforgeability

If there exists a polynomial-time adversary A that can crack the proposed attribute-based strong designated verifier signature scheme based on elliptic curve cryptography with a non-negligible advantage *ε*, then challenger C can solve the problem of the elliptic curve discrete logarithm problem (ECDLP) with a non-negligible probability.

*Proof*: The finite field *GF*(*p*) of order *p* is chosen, *E* is an elliptic curve defined on *GF*(*p*), and the system chooses the point *G* as the base point of this elliptic curve.

*1*. *Init*. Adversary A selects the attribute set ${W_{A}}^{*}$ of the signer and the attribute set ${W_{B}}^{*}$ owned by the designated verifier to be challenged, and sends them to challenger C.
*2*. *Setup*. challenger C selects a security parameter *k* and simulates the generation of public parameters as follows.
  Randomly selects $x\in {Z_{p}}^{*}$, calculate *P*_*pub*_ = *xG*. For each attribute *i*∈*U*, randomly select the secret value $z_{i}\in {Z_{p}}^{*}$ and compute *h*_*i*_ = *z*_*i*_*G*. Let $H:{\left\{0,1\right\}}^{*}\to {Z_{p}}^{*}$ be a secure cryptographic hash function. Challenger C generates the public parameter *params* = {*p*,*G*,*H*,*h*_1_,*h*_2_,⋯,*h*_*n*_,*P*_*pub*_} and the master key *MSK* = {*x*_1_,*z*_1_,*z*_2_,⋯*z*_*n*_} to adversary A.
*3*. *Queries*. Adversary A can perform polynomial times of key extraction queries, signature queries, and verify queries to challenger C.
  - Key extraction queries. Adversary A sends the access structure *T*_*A*_ satisfying $T_{A}({W_{A}}^{*})\neq 1$ to challenger C. Challenger C randomly selects $r_{A_{1}},r_{A_{2}},\ldots ,r_{A_{t}}\in {Z_{p}}^{*}$, constructs vector ${\vec{v}}_{A}=(x,r_{A_{2}},r_{A_{3}},\ldots ,r_{A_{t}})$, computes $\lambda_{A_{i}}={\vec{L}}_{A_{i}}\cdot {\vec{v}}_{A}$ for each row *i*∈[1,*S*_*A*_] of the LSSS matrix *L*_*A*_, and then computes the private key of the signer as $d_{A_{i}}=\lambda_{A_{i}}+z_{\rho_{A}(i)}$ and the public key of the signer as $PK_{A}={\left\{D_{A_{i}}\right\}}_{i\in [1,s_{A}]}$.

Adversary A sends the access structure *T*_*B*_ satisfying $T_{B}({W_{B}}^{*})\neq 1$ to challenger C. Challenger C randomly selects $r_{B_{1}},r_{B_{2}},\ldots ,r_{B_{t}}\in {Z_{p}}^{*}$ and constructs the vector ${\vec{v}}_{B}=(x,r_{B_{2}},r_{B_{3}},\ldots ,r_{B_{t}})$. For each row *i*∈[1,*S*_*B*_] of the LSSS matrix *L*_*A*_, compute $\lambda_{B_{i}}={\vec{L}}_{B_{i}}\cdot {\vec{v}}_{B}$. Compute the private key of the verifier as $d_{B_{i}}=\lambda_{B_{i}}+z_{\rho_{B}(i)}$ and the public key of the verifier as $PK_{B}={\left\{D_{B_{i}}\right\}}_{i\in [1,s_{B}]}$.

- Signature queries. Adversary A sends the attribute set *W*_*A*_ of the signer, message *M*, and verifier attribute set *W*_*B*_ of the verifier to challenger C. Challenger C signs message *M* according to the signature step.

If the attribute set *W*_*A*_ satisfies the access structure *T*_*A*_, then one can obtain a set of constants $\left\{\omega_{A_{i}}\in {Z_{p}}^{*},i\in \varpi_{A}\right\}$ such that $\sum_{i\in \varpi_{A}}\omega_{A_{i}}L_{A_{i}}=(1,0,\ldots ,0)$, where $\varpi_{A}=\{i\in [1,s_{A}]:\rho_{A}(i)\in W_{A}\}$. Randomly select $e\in {Z_{p}}^{*}$ and calculate *R* = *eM*, *r* = *H*(*R*), randomly select $k_{1}\in {Z_{p}}^{*}$ and calculate $s=k_{1}-r\sum_{i\in \varpi_{A}}d_{A_{i}}\omega_{A_{i}}$.

If the attribute set *W*_*B*_ of the verifier satisfies the access structure *T*_*B*_, then a set of constants $\left\{\omega_{B_{i}}\in {Z_{p}}^{*},i\in \varpi_{B}\right\}$ can be found in polynomial time such that $\sum_{i\in \varpi_{B}}\omega_{B_{i}}L_{B_{i}}=(1,0,\ldots ,0)$, where $\varpi_{B}=\{i\in [1,s_{B}]:\rho_{B}(i)\in W_{B}\}$.

Challenger C picks $k_{2}\in {Z_{p}}^{*}$ randomly and computes $V=k_{2}\sum_{i\in \varpi_{B}}D_{B_{i}}$ and $Z=k_{1}\sum_{i\in \varpi_{B}}D_{B_{i}}+k_{2}G$.

Challenger C sends the signature *σ* = (*R*,*s*,*V*,*Z*) to adversary A.

- Verify queries. Adversary A sends signature *σ* = (*R*,*s*,*V*,*Z*) to challenger C. Challenger C verifies the signature according to the verification algorithm as follows.

Challenger C computes *r* = *H*(*R*) and verifies that equation

$$Z=\sum_{i\in \varpi_{B}}d_{B_{i}}[sG+r(P_{pub}+\sum_{i\in \varpi_{A}}h_{A_{i}}\omega_{A_{i}})]+{\left(\sum_{i\in \varpi_{B}}d_{B_{i}}\right)}^{-1}V.$$ (4)

If it holds, then challenger C returns "1" to adversary A. Otherwise, it returns "0".

*4*. *Forgery*. Adversary A forges the signature σ* of message M*, corresponding to the attribute set of the signer as ${W_{A}}^{*}$, and specifies the attribute set of the verifier as ${W_{B}}^{*}$ to send to challenger C.

Challenger C first finds the reconstructed constant sets $\left\{\omega_{A_{i}}\in {Z_{p}}^{*},i\in \varpi_{A}\right\}$ and $\left\{\omega_{B_{i}}\in {Z_{p}}^{*},i\in \varpi_{B}\right\}$ based on the attribute sets ${W_{A}}^{*}$ and ${W_{B}}^{*}$ provided by adversary A. The constant sets satisfy the reconstruction of $\sum_{i\in \varpi_{A}}\omega_{A_{i}}L_{A_{i}}=(1,0,\ldots ,0)$ and $\sum_{i\in \varpi_{B}}\omega_{B_{i}}L_{B_{i}}=(1,0,\ldots ,0)$.

Then replaying with the same parameters and choosing a different hash function *H*_1_(∙), challenger C obtains another legal signature σ*′ for M* according to the forking lemma. Thus both σ* and σ*′ satisfy the verification equation, then there are the following equations

$$Z=\sum_{i\in \varpi_{B_{i}}}d_{B_{i}}[s^{*}G+r^{*}(P_{pub}+\sum_{i\in \varpi_{A}}h_{A_{i}}\omega_{A_{i}})]+{\left(\sum_{i\in \varpi_{B}}d_{B_{i}}\right)}^{-1}V$$ (5)

$$Z=\sum_{i\in \varpi_{B_{i}}}d_{B_{i}}[s^{*\prime }G+r^{*\prime }(P_{pub}+\sum_{i\in \varpi_{A}}h_{A_{i}}\omega_{A_{i}})]+{\left(\sum_{i\in \varpi_{B}}d_{B_{i}}\right)}^{-1}V$$ (6)

Subtract the two formulas to get:

$$0=\sum_{i\in \varpi_{B}}d_{B_{i}}[\left(s^{*}-s^{*\prime })G+(P_{pub}+\sum_{i\in \varpi_{A}}h_{A_{i}}\omega_{A_{i}})(r^{*}-r^{*\prime }\right)]$$

$$(s^{*}-s^{*\prime })G+(r^{*}-r^{*\prime })P_{pub}+(r^{*}-r^{*\prime })\sum_{i\in \varpi_{A}}h_{A_{i}}\omega_{A_{i}}=0(r^{*\prime }-r^{*})P_{pub}=(s^{*}-s^{*\prime })G+(r^{*}-r^{*\prime })\sum_{i\in \varpi_{A}}h_{A_{i}}\omega_{A_{i}}$$

$$(r^{*\prime }-r^{*})xG=(s^{*}-s^{*\prime })G+(r^{*}-r^{*\prime })\sum_{i\in \varpi_{A}}z_{i}G\omega_{A_{i}}$$

Since challenger C knows the process of signature generation and verification, it can calculate $x=[(s^{*}-s^{*\prime })+(r^{*}-r^{*\prime })\sum_{i\in \varpi_{A}}z_{i}\omega_{A_{i}}]{\left(r^{*\prime }-r^{*}\right)}^{-1}$.

Thus challenger C outputs *x* as a solution to the discrete logarithm problem, that is, if adversary A can successfully forge the attribute-based strongly designated verifier signature equal to cracking the elliptic curve discrete logarithm problem (ECDLP). Due to the fact that the elliptic curve discrete logarithm problem is a challenge based on the elliptic curve public key cryptosystem, no adversary A wins this game by a non-negligible advantage in polynomial time. The scheme satisfies unforgeability.

#### 4.2.4. Privacy non-transmissibility

Our efficient attribute-based strong designated verifier signature scheme based on elliptic curve cryptography satisfies privacy non-transmissibility.

*Proof*: The game between adversary A and challenger C is as follows.

1. *Init*. Adversary A selects the attribute set ${W_{A}}^{*}$ of the signer and the attribute set ${W_{B}}^{*}$ owned by the designated verifier to be challenged, and sends them to challenger C.
2. *Setup*. challenger C selects a security parameter *k* and simulates the generation of public parameters as follows.
  Randomly selects $x\in {Z_{p}}^{*}$, calculate *P*_*pub*_ = *xG*. For each attribute *i*∈*U*, randomly select the secret value $z_{i}\in {Z_{p}}^{*}$ and compute *h*_*i*_ = *Z*_*i*_*G*. Let $H:{\left\{0,1\right\}}^{*}\to {Z_{p}}^{*}$ be a secure cryptographic hash function. Challenger C generates the public parameter *params* = {*p*,*G*,*H*,*h*_1_,*h*_2_,⋯*h*_*n*_,*P*_*pub*_} and the master key *MSK* = {*x*,*z*_1_,*z*_2_,⋯,*z*_*n*_} to adversary A.
3. *Queries*. Adversary A can perform polynomial times of key extraction queries, signature queries, and verify queries to challenger C.
  - Key extraction queries. Adversary A sends the access structure *T*_*A*_ satisfying $T_{A}({W_{A}}^{*})\neq 1$ to challenger C. Challenger C randomly selects $r_{A_{1}},r_{A_{2}},\ldots ,r_{A_{t}}\in {Z_{p}}^{*}$, constructs vector ${\vec{v}}_{A}=(x,r_{A_{2}},r_{A_{3}},\ldots ,r_{A_{t}})$, computes $\lambda_{A_{i}}={\vec{L}}_{A_{i}}\cdot {\vec{v}}_{A}$ for each row *i*∈[1,*S*_*A*_] of the LSSS matrix *L*_*A*_, and then computes the private key of the signer as $d_{A_{i}}=\lambda_{A_{i}}+z_{\rho_{A}(i)}$ and the public key of the signer as $PK_{A}={\left\{D_{A_{i}}\right\}}_{i\in [1,s_{A}]}$.

Adversary A sends the access structure *T*_*B*_ satisfying $T_{B}({W_{B}}^{*})\neq 1$ to challenger C. Challenger C randomly selects $r_{B_{1}},r_{B_{2}},\ldots ,r_{B_{t}}\in {Z_{p}}^{*}$ and constructs the vector ${\vec{v}}_{B}=(x,r_{B_{2}},r_{B_{3}},\ldots ,r_{B_{t}})$. For each row *i*∈[1,*S*_*B*_] of the LSSS matrix *L*_*A*_, compute $\lambda_{B_{i}}={\vec{L}}_{B_{i}}\cdot {\vec{v}}_{B}$. Compute the private key of the verifier as $d_{B_{i}}=\lambda_{B_{i}}+z_{\rho_{B}(i)}$ and the public key of the verifier as $PK_{B}={\left\{D_{B_{i}}\right\}}_{i\in [1,s_{B}]}$.

- Signature queries. Adversary A sends the attribute set *W*_*A*_ of the signer, message *M*, and verifier attribute set *W*_*B*_ of the verifier to challenger C. Challenger C signs message *M* according to the signature step.

If the attribute set *W*_*A*_ satisfies the access structure *T*_*A*_, then one can obtain a set of constants $\left\{\omega_{A_{i}}\in {Z_{p}}^{*},i\in \varpi_{A}\right\}$ such that $\sum_{i\in \varpi_{A}}\omega_{A_{i}}L_{A_{i}}=(1,0,\ldots ,0)$, where $\varpi_{A}=\{i\in [1,s_{A}]:\rho_{A}(i)\in W_{A}\}$. Randomly select $e\in {Z_{p}}^{*}$ and calculate *R* = *eM*, *r* = *H*(*R*), randomly select $k_{1}\in {Z_{p}}^{*}$ and calculate $s=k_{1}-r\sum_{i\in \varpi_{A}}d_{A_{i}}\omega_{A_{i}}$.

If the attribute set *W*_*B*_ of the verifier satisfies the access structure *T*_*B*_, then a set of constants $\left\{\omega_{B_{i}}\in {Z_{p}}^{*},i\in \varpi_{B}\right\}$ can be found in polynomial time such that $\sum_{i\in \varpi_{B}}\omega_{B_{i}}L_{B_{i}}=(1,0,\ldots ,0)$, where $\varpi_{B}=\{i\in [1,s_{B}]:\rho_{B}(i)\in W_{B}\}$.

Challenger C picks $k_{2}\in {z_{p}}^{*}$ randomly and computes $V=k_{2}\sum_{i\in \varpi_{B}}D_{B_{i}}$ and $Z=k_{1}\sum_{i\in \varpi_{B}}D_{B_{i}}+k_{2}G$.

Challenger C sends the signature *σ* = (*R*,*s*,*V*,*Z*) to adversary A.

- Verify queries. Adversary A sends signature *σ* = (*R*,*s*,*V*,*Z*) to challenger C. Challenger C verifies the signature according to the verification algorithm as follows.

Challenger C computes *r* = *H*(*R*) and verifies that equation

$$Z=\sum_{i\in \varpi_{B}}d_{B_{i}}[sG+r(P_{pub}+\sum_{i\in \varpi_{A}}h_{A_{i}}\omega_{A_{i}})]+{\left(\sum_{i\in \varpi_{B}}d_{B_{i}}\right)}^{-1}V.$$ (7)

If it holds, then challenger C returns "1" to adversary A. Otherwise, it returns "0".

4. *Challenge*. For any message *M*′∈{0,1}*, challenger C randomly selects $k\in {Z_{p}}^{*}$, computes $Z=k\sum_{i\in \varpi_{B}}D_{B_{i}}$, and compute

$$r=H(R),\;s=k,\;V=-r{\left(\sum_{i\in \varpi_{B}}d_{B_{i}}\right)}^{2}(P_{pub}+\sum_{i\in \varpi_{A}}h_{A_{i}}\omega_{A_{i}}),$$ (8)

to generate the simulated signature *σ*′ = (*R*,*s*,*V*,*Z*).

$$\sum_{i\in \varpi_{B}}d_{B_{i}}[sG+r(P_{pub}+\sum_{i\in \varpi_{A}}h_{A_{i}}\omega_{A_{i}})]+{\left(\sum_{i\in \varpi_{B}}d_{B_{i}}\right)}^{-1}V$$

$$=s(\sum_{i\in \varpi_{B}}d_{B_{i}}G)+r\sum_{i\in \varpi_{B}}d_{B_{i}}(P_{pub}+\sum_{i\in \varpi_{A}}h_{A_{i}}\omega_{A_{i}})-{\left(\sum_{i\in \varpi_{B}}d_{B_{i}}\right)}^{-1}[r{\left(\sum_{i\in \varpi_{B}}d_{B_{i}}\right)}^{2}(P_{pub}+\sum_{i\in \varpi_{A}}h_{A_{i}}\omega_{A_{i}})]$$

$$=s(\sum_{i\in \varpi_{B}}d_{B_{i}}G)+r\sum_{i\in \varpi_{B}}d_{B_{i}}(P_{pub}+\sum_{i\in \varpi_{A}}h_{A_{i}}\omega_{A_{i}})-r\sum_{i\in \varpi_{B}}d_{B_{i}}(P_{pub}+\sum_{i\in \varpi_{A}}h_{A_{i}}\omega_{A_{i}})$$

$$=s(\sum_{i\in \varpi_{B}}d_{B_{i}}G)$$

$$=k\sum_{i\in \varpi_{B}}D_{B_{i}}$$

=Z

This signature also enables the verification equation

$$Z=\sum_{i\in \varpi_{B}}d_{B_{i}}[sG+r(P_{pub}+\sum_{i\in \varpi_{A}}h_{A_{i}}\omega_{A_{i}})]+{\left(\sum_{i\in \varpi_{B}}d_{B_{i}}\right)}^{-1}V\;\mathrm{to}\;\mathrm{hold}.$$

The signature *σ*′simulated by challenger C is indistinguishable from the signature *σ* generated by the signer. The probability that adversary A can determine in polynomial time whether signature *σ* was generated by the signer or challenger C is negligible.

## 5. Efficiency analysis

This section analyses the efficiency of an efficient attribute-based strong designated verifier signature scheme based on elliptic curve cryptography. Table 1 compares our scheme with several other typical attribute-based strong designated verifier signature schemes in terms of access structure, access policy, private key and signature size, signature computation and verification efficiency. Here we have selected four typical attribute-based strong designated verifier signature schemes *SABSDVS* [47], *FABSDVS* [48], *ZABDCS* [52] and *TABSDVS* [49].

**Table 1:** Comparision of schemes.

Scheme	Access structure	Access policy	Private key sizes (|*G*|)	Signature sizes (|*G*|)	Signature computation (*T*_exp_)	Verification computation (*T*_*bp*_)
*SABSDVS*	Threshold access structure	*SP*	*w*	1	3	2
*FABSDVS*	Access tree	*SP*	3*w*	2*w*	5	5
*ZABDCS*	Monotonic boolean circuit	*KP*	*w*+2*q*_1_+3*q*_2_+1	*w*+1	*n*+2	2
*TABSDVS*	Lagrangian interpolation method	*SP*	*w*	4	2	4
Our scheme	LSSS matrix	*KP*	*w*	4	0	0

In Table 1, the meaning of each symbol is as follows: *w* denotes the number of attributes, *n* is the overall number of attributes, *s* denotes the number of attributes of the visitor, |*G*| denotes the length of the group *G* element, *T*_exp_ denotes the time of modulo power operation, *T*_*bp*_ denotes the time required for bilinear pairwise operation, *q*_1_ is the number of monotonic Boolean circuits or gates, and *q*_2_ is the number of monotonic Boolean circuits and gates. See S1 File.

We analyze the efficiency of the above schemes in terms of the access structure, the number of operations, and the length of the secret key and signature. The algorithm execution time consumption is mainly distributed in exponential (*T*_exp_) and bilinear pairing (*T*_*bp*_) operations, so the table mainly analyzes these two operations.

As can be seen in Table 1, the scheme has no bilinear pairing operations for both signature and verification calculations compared to other comparison schemes. One bilinear pairing operation on the same curve is 2–3 times more than the scalar multiplication [3]. Therefore, it is more efficient to use scalar multiplication on elliptic curves instead of bilinear pairing operations to construct attribute-based strong designated verifier signature schemes in the signing and verification process. In addition, most of the access structures relied on by the existing attribute-based strong designated verifier signature schemes are threshold access structures or access tree structures, which have many limitations in policy expression. The LSSS matrix is stronger in access policy expression and can express any access policy, including "And" gate, "Or" gate and threshold, with flexible access structure [57]. The new scheme uses the LSSS access structure to construct an efficient attribute-based strong designated verifier signature scheme based on elliptic curve cryptography, which is more efficient in both signature generation and verification. It is more efficient than the existing attribute-based strongly designated verifier signature schemes. Also, the signature generation process uses concatenated summation operations to make the signature length fixed. Of course, the limitations of the breadth of the literature search may have led to omissions in the comparison scheme, and we will try to improve this in future research work.

## 6. Conclusions

It is a hot research topic in the field of cryptography to improve the efficiency and security of attribute-based strongly designated verifier signature schemes as much as possible. Most of the existing attribute-based strong designated verifier signature schemes involve complex bilinear pairing operations, which makes the overall scheme inefficient. To address this problem, in this paper, an efficient attribute-based strong designated verifier signature scheme based on elliptic curve cryptography is proposed and analyzed for its security. In Section 3, we present some background knowledge and optimize the security model of an attribute-based strong designated verifier signature scheme to facilitate better understanding of the newly proposed scheme. In Section 4, we give our construction of a new efficient attribute-based strong designated verifier signature scheme based on elliptic curve cryptography. The security of the proposed scheme is analyzed under the difficulty of the elliptic curve discrete logarithm problem (ECDLP) on which the elliptic curve cryptography is based. The new scheme uses scalar multiplication on elliptic curves, which is more lightweight, instead of bilinear pairing operations, which have a higher computational overhead [58, 59]. This reduces the computational overhead in the signature and verification process, making the scheme more suitable for cloud endpoint scenarios and resource-constrained devices. The new scheme replaces the bilinear pairing operation with scalar multiplication on elliptic curves providing a new idea for the study of attribute-based strong designated verifier signature schemes. Meanwhile, our scheme uses LSSS matrix to represent the access structure. LSSS takes advantage of the linear secret sharing scheme’s secret reconfigurable nature to reconstruct the secret without recursive operations, improves the signature and efficiency of attribute-based signature schemes, and makes the policy expression more flexible. Compared with several attribute-based strong designated verifier signature schemes in Section 5, the new scheme designed in this paper not only improves the efficiency of access policy expression, but also achieves the signature length independent of the number of signer attributes. The new scheme has greater advantages in terms of computational efficiency and storage space.

## Supporting information

S1 File (DOCX)
