# Supplementary material for: Efficient attribute-based strong designated verifier signature scheme based on elliptic curve cryptography
Source: PLoS One. 2024 May 9;19(5):e0300153. doi: 10.1371/journal.pone.0300153 (PMC11081367; doi:10.1371/journal.pone.0300153)
Supplement: S1 File — (DOCX) [file pone.0300153.s001.docx]

**S1 File.**

The efficiency of our scheme,$SABSDVS$[47],$FABSDVS$[48],$ZABDCS$[52] and$TABSDVS$[49]are analyzed in terms of access structure, number of operations, secret key, and signature lengths. The algorithm execution time consumption is mainly distributed in exponential$\left(T_{\exp}\right)$and bilinear pairing$\left(T_{bp}\right)$operations.

**Comparision of Schemes.**

| Scheme | Access structure | Access policy | Private  key sizes$\left(\left\vert G\right\vert\right)$ | Signature sizes $\left(\left\vert G\right\vert\right)$  | Signature computation$\left(T_{\exp}\right)$ | Verification computation$\left(T_{bp}\right)$ | |  |
| --- | --- | --- | --- | --- | --- | --- | --- | --- |
| $SABSDVS$ | Threshold access structure | $SP$ | $w$ | $1$ | $3$ | | $2$ | |
| $FABSDVS$ | Access  tree | $SP$ | $3w$ | $2w$ | $5$ | | $5$ | |
| $ZABDCS$ | Monotonic boolean circuit | $KP$ | $w+2q_{1}+3q_{2}+1$ | $w+1$ | $n+2$ | | $2$ | |
| $TABSDVS$ | Lagrangian interpolation method | $SP$ | $w$ | $4$ | $2$ | | $4$ | |
| Our scheme | LSSS  matrix | $KP$ | $w$ | $4$ | $0$ | | $0$ | |

^1^$w$: denotes the number of attributes,

^2^$n$: is the overall number of attributes,

^3^$s$: denotes the number of attributes of the visitor,

^4^$\left|G\right|$: denotes the length of the group$G$element,

^5^$T_{\exp}$: denotes the time of modulo power operation,

^6^$T_{bp}$: denotes the time required for bilinear pairwise operation,

^7^$q_{1}$: is the number of monotonic Boolean circuits or gates,

^8^$q_{2}$: is the number of monotonic Boolean circuits and gates.
